# A Potential Central Hub of Histamine in the Microbiota–Gut–Joint Axis in Rheumatoid Arthritis: Mechanisms and Translational Implications

**DOI:** 10.3390/ijms27052315

**Published:** 2026-03-01

**Authors:** Yiqing Kong, Yu Deng, Yuan Liu, Yuge Han, Yuandan Zhang, Zihan Qi, Menglei Cao, Yingying Li, Yu Du, Yan Jin, Jie Yu

**Affiliations:** 1 The Second Clinical Medical College, Zhejiang Chinese Medical University, Hangzhou 310053, China; 19883232866@163.com (Y.K.); 202212210916004@zcmu.cn (Y.H.); lmszlyy_0522@outlook.com (Y.L.); jinyan1003@hotmail.com (Y.J.); 2 College of Life Science, Zhejiang Chinese Medical University, Hangzhou 310053, China; 202312211702041@zcmu.edu.cn (Y.D.); 202312213903043@zcmu.edu.cn (Y.L.); 19884859590@163.com (Y.Z.); 3 Key Laboratory of Neuropharmacology and Translational Medicine of Zhejiang Province, College of Basic Medical Science, Zhejiang Chinese Medical University, Hangzhou 310053, China; 18039672710@163.com (Z.Q.); a19932136689@163.com (M.C.)

**Keywords:** rheumatoid arthritis, microbiota–gut–joint axis, histamine, histidine decarboxylase, histamine receptors, immunometabolism, fibroblast-like synoviocytes

## Abstract

Rheumatoid arthritis (RA) is a chronic autoimmune disease characterized by pain, persistent synovial inflammation, progressive joint destruction, and systemic immune dysregulation. Increasing evidence has revealed that the microbiota–gut–joint axis represents a crucial communication network linking intestinal dysbiosis to aberrant immune responses in RA. Among the diverse gut-derived metabolites implicated in this axis, we propose that histamine may act as a central signaling node linking microbial alterations to joint inflammation. Both host- and microbiota-derived histamine, synthesized via histidine decarboxylase (HDC), regulate immune and stromal cell activity within the joint microenvironment through histamine receptors H1R, H2R, and H4R. In addition, histamine interacts with other microbial metabolites—such as short-chain fatty acids (SCFAs) and tryptophan derivatives—forming an intricate metabolic–inflammatory network that amplifies fibroblast-like synoviocyte activation, osteoclastogenesis, and chronic inflammation. Despite accumulating evidence supporting the immunomodulatory role of histamine, the precise molecular mechanisms mediating its crosstalk with microbial and host immune pathways remain incompletely defined. This review provides a comprehensive overview of histamine-mediated regulation within the microbiota–gut–joint axis, emphasizing its interplay with other microbial metabolites and its contribution to RA pathogenesis. A deeper understanding of this histamine-centered microbiota–gut–joint axis will help elucidate its mechanistic role in immune dysregulation and may ultimately inform future strategies for restoring immune balance and preventing joint damage in RA.

## 1. Introduction

Rheumatoid arthritis (RA) is a chronic, systemic inflammatory autoimmune disease that primarily affects synovial joints, leading to extensive joint damage, pain, and loss of function [1]. The global prevalence of RA ranges from 0.5% to 2%, with reported rates in China between 0.30% and 0.42%. Middle-aged women, smokers, and individuals with a family history of RA are at higher risk [2]. Clinically, RA is characterized by progressive, symmetrical inflammation of affected joints, resulting in cartilage degradation, bone erosion, and functional disability, and may ultimately lead to joint deformity or even death [3]. Although RA typically begins with a limited number of small joints, disease progression often involves larger joints such as the shoulders, elbows, knees, and ankles, causing swelling and pain that may ease with physical activity [4]. Current treatment strategies for RA rely primarily on pharmacotherapy, including nonsteroidal anti-inflammatory drugs (NSAIDs), glucocorticoids (GCs), conventional disease-modifying antirheumatic drugs (DMARDs), and biologics [5]. In cases of severe joint damage, joint replacement surgery is often employed as an adjunctive approach [5]. Notably, the proportion of patients achieving clinical remission increased from approximately 6% in 2004 to 32% in 2015, coinciding with the implementation of targeted therapy strategies and the introduction of biologic DMARDs [6]. Registry data from 2016 further demonstrated that over half of patients were able to achieve sustained clinical remission (DAS28-ESR < 2.6) within 6 months of diagnosis [7,8]. However, these advances mask persistent challenges: many patients still experience progressive joint damage or intolerable adverse effects, and the burden of frequent medical visits and monitoring remains significant. Furthermore, seronegative patients—traditionally considered to have a better prognosis—have not equally benefited from these advances, highlighting differences in underlying disease mechanisms.

The pathogenesis of joint destruction in RA is multifactorial and complex. Synovial inflammation is a hallmark, characterized by hyperplasia of synovial lining cells and infiltration of immune cells, leading to the formation of invasive pannus tissue that progressively erodes cartilage and bone [9]. A persistent imbalance in pro-inflammatory cytokines, such as TNF-α and IL-6, fuels this destructive process [10]. Over time, chronic inflammation can cause irreversible joint damage and deformity, underscoring the importance of early intervention [9]. In addition, pain, as another dominant clinical manifestation of RA, is equally complex and remains poorly understood. Although inflammatory cytokines contribute to the sensitization of joints’ nociceptors and pain transmission, they do not fully explain the chronic pain experienced by many patients [11]. This renders conventional anti-inflammatory therapies insufficient for complete pain relief in RA patients.

Growing evidence suggests that dysbiosis of the gut microbiota is a key environmental factor that triggers the dysregulation of both innate and adaptive immune responses, contributing to the onset of RA arthritic pain [12]. This plays a crucial role in maintaining immune homeostasis, regulating inflammatory responses, and influencing bone metabolism. Under physiological conditions, a balanced gut microbiome supports immune tolerance and prevents excessive inflammation, contributing to joint health. However, when dysbiosis occurs, microbial alterations can disrupt immune equilibrium, leading to local and systemic inflammatory responses that impact joint integrity. Significant alterations have been identified in the gut microbiota composition of RA patients, characterized by an overrepresentation of pro-inflammatory bacteria (e.g., *Prevotella copri*) and a depletion of beneficial microbes (e.g., *Faecalibacterium prausnitzii*). Thus, the “gut-joint axis”, a critical area of investigation in the pathogenesis of RA, constitutes intricate bidirectional interactions between the gut microbiota and the musculoskeletal system, as well as the dynamic destruction of related joints.

Gut-derived microbial metabolites, including short-chain fatty acids (SCFAs), tryptophan metabolites, histamine, etc., play a critical role in mediating the effects of the gut–joint axis. These metabolites influence joint inflammation, fibroblast-like synoviocyte (FLS) activation, and osteoclastogenesis, ultimately contributing to synovial hyperplasia, cartilage destruction, and bone erosion in RA. Among them, histamine has emerged as a key immunomodulatory molecule with both local and systemic effects relevant to RA. In addition to its established roles in allergic inflammation, histamine exerts diverse regulatory effects on immune responses and tissue remodeling, mediated through four G protein-coupled receptors (H1R–H4R). Histamine H1R and H2R have been identified on human monocytes and macrophages, where they contribute to the regulation of joints’ inflammatory responses [13,14]. Histamine regulates the differentiation and function of osteoclasts and osteoblasts through multiple signaling cascades, including the RANKL-RANK pathway [15]. Of particular interest in RA is the histamine H4 receptor, which is expressed in synovial tissues, vasculature, and FLSs. Specifically, the differential engagement of H1R and H2R influences osteoclast maturation and osteoblast activity [16], while H4R exerts a dominant effect under inflammatory conditions, promoting osteoclast formation and contributing to joint destruction [15]. These findings underscore the widespread expression of histamine receptors on key effector cells of RA—including FLSs, macrophages, osteoclasts, and osteoblasts—and further highlight the potential role of histaminergic signaling in mediating the effects of the gut–joint axis.

Moreover, histamine does not operate in isolation but is intricately intertwined with other gut microbiota-derived metabolic pathways, particularly those involving SCFAs. These pathways modulate histamine synthesis and receptor activity, thereby influencing the inflammatory microenvironment in RA. This interdependent mechanism suggests that histamine may act as a central mediator of both microbiota- and host-derived signals within the gut–joint axis. Given this integrated framework, targeting histamine metabolism through microbiota-directed strategies emerges as a promising therapeutic approach.

This review aims to summarize current knowledge of the mechanisms governing the microbiota–gut–joint axis in RA, with particular emphasis on histamine-mediated interactions between gut microbial metabolites and joint dysfunction. We focus on the roles of histamine, SCFAs, and tryptophan metabolites in modulating immune responses, synovial inflammation, and bone remodeling. By elucidating the complex crosstalk between gut microbiota and histaminergic signaling, this review provides new insights into the pathophysiology of RA and potential histamine-targeted therapeutic strategies.

## 2. The Microbiota–Gut–Joint Axis in RA

In RA patients, the gut microbiota exhibits significant community alterations compared to healthy individuals, characterized by reduced microbial diversity and shifts in the relative abundance of specific taxa. At the phylum level, RA patients show a marked increase in *Verrucomicrobacteria* and *Proteobacteria* while the abundance of *Bacteroidetes* and *Firmicutes* is significantly decreased [17]. At the genus level, there is a notable increase in the relative abundance of genera such as *Lactobacillus*, *Streptococcus*, *Akkermansia*, *Ruminococcus* II, *Klebsiella*, *Escherichia*, *Eisenbergiella*, *Flavobacterium*, *Bacteroides*, and *Prevotella* in RA patients. Conversely, *Roseburia*, *Lachnoclostridium*, *Faecalibacterium*, *Clostridium*, *Dialister*, *Megamonas*, *Gemmiger*, *Parasutterella*, *Acetivibrio*, *Coprococcus*, *Anaerostipes*, *Enterococcus*, *Chromochromoin*, *Escherella*, *Serratia*, *Corynebacteriu* I, *Granulosa*, *Staphylococcus aureus*, *Gemella*, *Bifidobacterium*, and *Blautia* are significantly reduced [18,19,20,21]. The available evidences suggest that gut microbiome dysbiosis seems to aggravate RA by enhancing the immune response, thus increasing the inflammatory state of the articular environment.

Specifically, gut microbiome dysbiosis may induce higher levels of systemic lipopolysaccharides (LPS) and subsequent elevation of other proinflammatory factors, such as TNF-α, IL-1β, IL-6, and C-reactive protein (CRP), thus supposedly enhancing RA progression [22]. Five microbial phyla were found to be dominant in knees with inflammation: *Proteobacteria*, *Actinobacteria*, *Firmicutes*, *Fusobacteria*, and *Bacteroidetes*. In most studies, *Proteobacteria* was the most frequently identified phylum. Within *Proteobacteria*, *Escherichia* spp. [23] and *Pseudomonas* spp. [24] were commonly identified, alongside an increase in Gram-negative constituents in the microbial joint DNA [23,25,26,27]. Hammad et al. reported that the microbiome of nine healthy controls was predominated by *Proteobacteria* (83.5%) and *Firmicutes* (16.1%). The evidences agree with the present literature reporting *Proteobacteria phylum* as a major constituent of the gut microbiome [28,29] and its expansion as a possible actor in joint inflammation of RA.

The gut microbiota can directly induce joint inflammation through specific bacterial strains. *Prevotella copri*, a gut microbe closely associated with RA progression, has been detected in both the intestinal tract and synovial fluid (SF) of RA patients. Studies have shown that *P. copri* promotes Th17 cell activation and increases the production of pro-inflammatory cytokines such as IL-17, thereby amplifying inflammation and bone destruction in RA joints [30]. Beyond specific bacterial species, gut microbiota dysbiosis can also contribute to joint inflammation by disrupting the balance between pro-inflammatory and anti-inflammatory signals. This imbalance directly affects FLS, key mediators in RA pathogenesis that drive synovial tissue remodeling and the production of pro-inflammatory cytokines. Notably, RA patients exhibit a reduced abundance of *Faecalibacterium prausnitzii*, a gut commensal with anti-inflammatory properties. The depletion of this bacterium may impair immune regulation, leading to heightened FLS activity and exacerbated joint inflammation [31].

**Figure 1:**
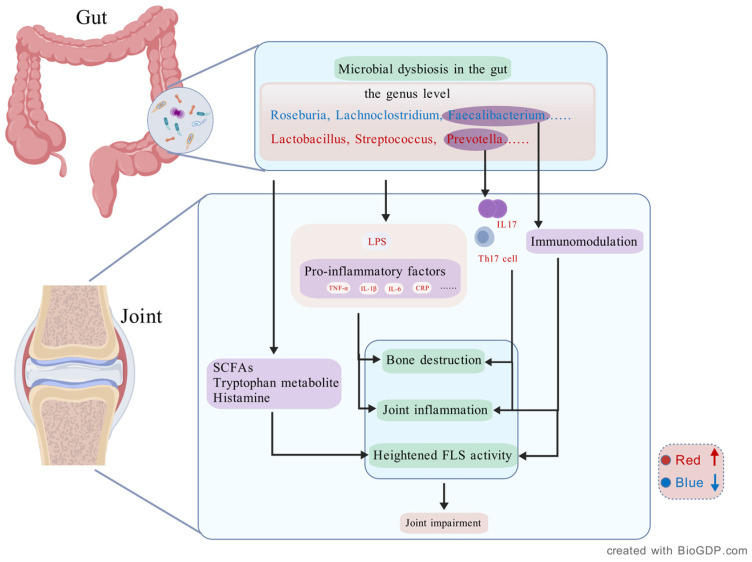
Microbiota–gut–joint axis and its contribution to RA pathogenesis. In RA, gut dysbiosis involves decreased levels of beneficial genera (*Roseburia*, *Lachnoclostridium*, *Faecalibacterium*) and enrichment of pro-inflammatory taxa (*Lactobacillus*, *Streptococcus*, *Prevotella*). This shift increases microbial metabolites and endotoxins like LPS, inducing cytokines (TNF-α, IL-1β, IL-6, CRP) that promote joint inflammation and bone loss. *Prevotella copri* facilitates Th17 differentiation and IL-17 production, aggravating synovial inflammation. Loss of *Faecalibacterium prausnitzii* weakens immune tolerance and elevates FLS activity. Microbial metabolites—SCFAs, tryptophan derivatives, and histamine—modulate bone destruction, joint inflammation and FLS activity, contributing to RA progression. Created with BioGDP.com [33].

In addition to synovial inflammation, gut microbiota dysbiosis plays a critical role in bone destruction—a hallmark of RA. Osteoclasts, the primary cells responsible for bone resorption, are highly regulated by inflammatory cytokines. Microbial dysbiosis has been associated with elevated levels of pro-inflammatory mediators that drive osteoclast differentiation, ultimately leading and exacerbating bone damage in RA patients [32]. Within this axis, gut microbiota alterations contribute to joint damage through multiple interconnected mechanisms, including the modulation of immune responses, activation of FLS, and promotion of osteoclastogenesis. Additionally, microbial metabolites such as SCFAs, tryptophan metabolites, and histamine play crucial roles in these processes by modulating inflammatory signaling, immune cell activity, and bone metabolism (Figure 1).

### 2.1. Dysbiosis, Gut Microbial Metabolites, and Joint Dysfunction in RA

Dysbiosis of the gut microbiota is a hallmark of RA and is closely associated with alterations in gut microbial metabolism. Key SCFA-producing bacterial taxa, including *Faecalibacterium* [34], *Ruminococcus*, *Ruminiclostridium* [35], *Megasphaera* [36,37], *Clostridiales* [38], *Lactobacillus paracasei* [38], *Lachnospira* [39], and *Bifidobacterium* [40], are significantly changed in RA patients. Among these, *Faecalibacterium* [41], *Megasphaera* [40], and *Bifidobacterium* [42,43] are particularly depleted, whereas the abundance of *Clostridiales* is often increased in RA patients [44,45]. Interestingly, the abundance of specific probiotics, such as *Lachnospira* [46], increases with RA disease activity. Dysbiosis in RA is characterized by decreased bacterial diversity, a reduction in Gram-negative bacteria (e.g., *Haemophilus* and *Prevotella*), and an increase in Gram-positive bacteria (e.g., *Streptococcus* and *Faecalibacterium*) [47]. Specifically, RA patients exhibit a depletion of butyrate-producing bacteria (e.g., *Faecalibacterium*) and an enrichment of butyrate-utilizing bacteria (e.g., *Prevotella*) [48]. These findings underscore a direct connection between gut microbiota composition, metabolites production, and immune regulation in RA. Importantly, some gut microbial imbalances can be partially restored following RA treatment remission, highlighting the dynamic relationship between microbiota composition, metabolites levels, and disease progression [49]. By modulating gut barrier integrity, immune responses, and osteoclast activity, SCFAs serve as a critical component of the gut–joint axis.

SCFAs, including acetate, propionate, and butyrate, play a pivotal role in joint health, exhibiting both protective and potentially detrimental effects in RA. While SCFAs are generally recognized as anti-inflammatory metabolites, their impact on bone metabolism and immune modulation is complex. Evidence suggests that SCFAs influence osteoclastogenesis, the process of osteoclast-driven bone resorption, which is central to RA-associated bone erosion. Lucas et al. reported that both direct SCFA supplementation and a high-fiber diet in mice resulted in increased systemic bone density, reduced bone resorption, and decreased osteoclast levels [50]. Moreover, SCFA treatment significantly attenuated arthritis severity, which was associated with systemic bone mass gain and notable downregulation of osteoclast-specific genes, including TRAF6 and NFATc1 [50]. Similarly, Kadono et al. reported that butyrate and propionate induced a metabolic shift toward glycolysis in osteoclasts and significantly inhibited TRAF6, a key component of osteoclastogenic signaling [51]. Additionally, butyrate has been shown to suppress osteoclast differentiation and mitigate RA-associated joint deformity by modulating histone deacetylase activity and altering KEGG pathways linked to osteoclast differentiation [48]. Furthermore, butyrate attenuates joint inflammation by inhibiting cytokine production in invariant natural killer T (iNKT) cells [52]. These findings highlight the crucial role of SCFAs in joint damage regulation.

**Figure 2:**
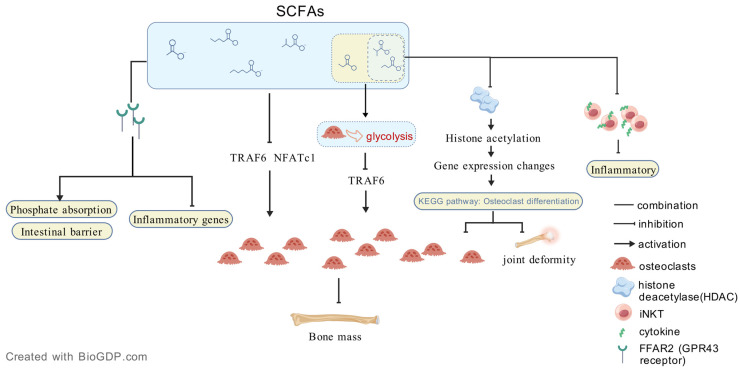
Mechanisms by which SCFAs regulate bone metabolism and inflammation in rheumatoid arthritis. SCFAs produced by gut microbiota regulate host immunity and bone homeostasis via multiple pathways. By binding to FFAR2 (GPR43), SCFAs promote phosphate absorption, enhance intestinal barrier integrity, and suppress inflammatory gene expression. They also reduce osteoclast differentiation by downregulating TRAF6 and NFATc1 signaling. Butyrate and propionate shift osteoclast metabolism toward glycolysis and markedly inhibit TRAF6, a key mediator of osteoclastogenesis. Butyrate also limits osteoclastogenesis and joint damage via HDAC inhibition and KEGG pathway modulation, and reduces inflammation by suppressing cytokine release from iNKT cells. Together, these actions of SCFAs preserve bone integrity and attenuate RA progression. Created with BioGDP.com [33].

Beyond their role in joint protection, SCFAs are essential for maintaining immune homeostasis. They reinforce gut barrier integrity by upregulating tight junction proteins and reducing zonulin levels, thereby improving gut permeability [53]. Additionally, SCFAs inhibit the nuclear factor κB (NF-κB) pathway [54,55], leading to the downregulation of pro-inflammatory mediators, including cytokines, chemokines, inducible nitric oxide synthase (iNOS), cyclooxygenase-2 (COX-2), adhesion molecules, and acute-phase proteins [56]. Moreover, SCFAs promote the differentiation of regulatory T cells (Tregs), which are essential for immune tolerance and suppression of autoimmune responses in RA [57,58]. SCFAs exert their biological effects primarily through binding to G protein-coupled receptors, such as free fatty acid receptor 2 (FFAR2) [59], which plays a key role in mediating their immunomodulatory and metabolic functions. By activating FFAR2, SCFAs facilitate phosphorus absorption, enhance intestinal barrier integrity, and suppress inflammatory gene expression in both the gut and bone marrow [60] (Figure 2).

In addition to SCFAs, Omega-3 Polyunsaturated Fatty Acids (PUFAs), particularly eicosapentaenoic acid (EPA) and docosahexaenoic acid (DHA), have been extensively studied for their anti-inflammatory effects in RA. These fatty acids regulate immune cell function by modulating inflammatory pathways, reducing the production of pro-inflammatory eicosanoids and cytokines, and inhibiting NF-κB activation [61]. Notably, omega-3 fatty acids enhance the abundance of SCFA-producing bacteria, suggesting a synergistic interaction between these two metabolite classes in controlling inflammation and joint damage in RA [62]. SCFAs, particularly butyrate, enhance the production of anti-inflammatory cytokines, and omega-3 fatty acids further contribute to inflammation resolution by giving rise to specialized pro-resolving mediators (SPMs), such as resolvins and protectins [63].

Omega-3 fatty acids can induce a reversible increase in SCFA-producing bacteria, further enhancing immune regulation within the gut–joint axis. This interplay underscores the complementary roles of omega-3 fatty acids and SCFAs in modulating RA pathology [62]. By influencing both gut microbial composition and systemic inflammation, SCFAs and omega-3 fatty acids contribute to immune homeostasis and potentially mitigate RA-associated joint damage.

Tryptophan (Trp) is an essential amino acid that is primarily metabolized through the kynurenine pathway (KP) and indole derivatives pathway, producing a range of bioactive metabolites. These metabolites play important roles in inflammation regulation and immune homeostasis, potentially influencing the pathogenesis of RA [38]. The KP is the primary metabolic route for tryptophan, with approximately 90–95% of dietary tryptophan being metabolized through this pathway [64,65]. This process is catalyzed by indoleamine 2,3-dioxygenase (IDO1, IDO2) and tryptophan 2,3-dioxygenase (TDO), generating various metabolites such as kynurenine (KYN), xanthurenic acid (XA), and quinolinic acid (QA), which are closely related to immune modulation and oxidative stress. Studies have shown that RA patients exhibit significantly lower serum Trp levels, while KYN and its downstream metabolites, such as XA and QA, are significantly elevated, indicating high activity of this pathway in RA patients. Moreover, baseline levels of 3-hydroxykynurenine (3-HK) and 3-hydroxyanthranilic acid (3-HAA) are decreased, reflecting the complex regulatory mechanisms of the KP metabolic dynamics [66].

In the FLS of RA patients, the expression of IDO1 is significantly increased, leading to tryptophan depletion and activation of immunosuppressive signaling pathways such as the GCN2 kinase pathway [67]. This local metabolic imbalance is closely associated with downregulation of the T cell receptor (TCR) ζ chain and T cell dysfunction. At the same time, elevated levels of quinolinic acid are detected in the SF of RA patients, a phenomenon not observed in healthy controls, suggesting that this metabolite may promote synovial inflammation and joint damage in RA. IDO1 plays a dual role in RA. On the one hand, by depleting tryptophan, IDO1 suppresses T cell proliferation and may exert an anti-inflammatory effect [68]. The enhanced IDO1 activity in RA patients is often associated with enhanced regulatory T cell (Treg) function, helping to control excessive immune activation and inflammation [69,70]. On the other hand, IDO1 metabolites such as QA and 3-HK have been shown to activate pro-inflammatory signaling pathways, increase oxidative stress, and exacerbate inflammation [69,71]. Studies have demonstrated that elevated IDO1 activity in RA patients correlates with disease severity, suggesting a potential pro-inflammatory role in the pathological process of RA [72].

In contrast to IDO1, IDO2 primarily regulates the T cell–B cell interface, with its expression being critical for autoantibody production and the maintenance of T cell helper functions [73,74]. Animal studies have shown that treatment with cell-permeable anti-IDO2 monoclonal antibodies significantly suppressed the onset of arthritis, indicating that IDO2 may be a potential therapeutic target for RA [75]. Furthermore, IDO1 and IDO2 are primarily distributed in both of peripheral tissues and the gut, where they play a crucial role in maintaining gut microbiota homeostasis and local immune balance. In RA, abnormal activity of IDO1 may indirectly exacerbate joint inflammation by affecting gut barrier function and microbiota metabolism [76,77]. Animal studies have also shown that IDO1 deficiency or inhibition leads to aggravated arthritis, further supporting its protective role in disease control [78]. However, while IDO1 and IDO2 exert multifaceted roles in RA pathogenesis, their contributions in RA remain highly context-dependent and incompletely understood.

In addition to the KP, the role of indole derivatives in RA pathogenesis has also gradually gained attention. Studies have found that in collagen-induced arthritis (CIA) rats, the levels of indole-3-acetic acid (IAA), indole-3-propionic acid (IPA), and indoleacetaldehyde (IAld) in serum were significantly reduced, suggesting that dysregulation of indole metabolism may be related to the pathogenesis of RA [79]. Among these metabolites, the decrease in IAA levels was the most pronounced, further supporting the potential link between indole derivatives and inflammation regulation [79]. IAA can activate the aryl hydrocarbon receptor (AhR) and its downstream signaling pathways (such as CYP1A1), regulating T cell differentiation and function, and balancing innate and adaptive immunity. IAA has been shown to enhance Treg cell differentiation and stabilize Foxp3 expression by reducing its ubiquitination and degradation via the AhR-TAZ-Tip60 signaling pathway, thus promoting an immunosuppressive environment, and alleviating synovial inflammation and joint damage in RA [4,80,81]. Moreover, in the CIA model, IAA treatment significantly reduced synovial inflammation and swelling in RA rats, increased the binding levels of Foxp3 and Tip60 in the synovium, and downregulated the expression of the Hippo signaling pathway-related factor TAZ, further confirming the anti-inflammatory effects of IAA and its correlation with AhR mediated immune regulatory pathways [4].

IPA is another important derivative of tryptophan metabolism by the gut microbiota, showing significant potential for antioxidation and anti-inflammation, which may make it a candidate for chronic inflammatory disease treatment. Studies have found that the level of IPA in RA patients is negatively correlated with disease activity score in 28 joints (DAS28) [82]. IPA significantly inhibits Th17 cell differentiation in environments favoring Th17 cell generation [82], and Th17 cells and their pro-inflammatory cytokine IL-17 are closely associated with the pathological development of RA [83]. As a potent AhR agonist, IPA upregulates Foxp3 expression by regulating Foxp3 gene promoter methylation, promoting Treg cell expansion and suppressing RA-associated immune dysregulation [84,85]. Furthermore, IPA reduces the levels of pro-inflammatory cytokine IL-17 while increasing the expression of anti-inflammatory cytokine IL-10, restoring immune cell cytokine balance, and further explaining its anti-arthritis effects [86,87,88]. Animal studies also support the joint-protective effects of IPA. In the CIA model, oral administration of IPA significantly reduced the severity of synovial inflammation and alleviated cartilage and bone erosion [82,86,87,89]. These findings suggest that IPA plays an important regulatory role in the pathology of RA synovium by modulating the Th17/Treg cell ratio and the secretion of related factors.

In RA patients, the levels of quinolinic acid are significantly elevated, enhancing the proliferation and metabolic activity of FLS, contributing to synovial hyperplasia and joint destruction [90]. Additionally, KP metabolites like KYN decrease mitochondrial respiratory capacity, inhibiting the osteogenic differentiation of human mesenchymal stem cells (hMSC) and promoting osteoclast maturation and differentiation via the RANKL signaling pathway, leading to bone resorption [65,91]. The KP also affects macrophage polarization, driving a pro-inflammatory phenotype in RA joints [13,71,92,93]. KYN metabolites like 3-HK promote the accumulation of reactive oxygen species (ROS) inside cells, further damaging mitochondrial DNA, inhibiting osteoblast activity, and hindering bone formation. Additionally, the interaction between tryptophan metabolism and SCFAs and gut microbiota may influence the pathogenesis of RA by regulating serotonin synthesis [94,95].

**Figure 3:**
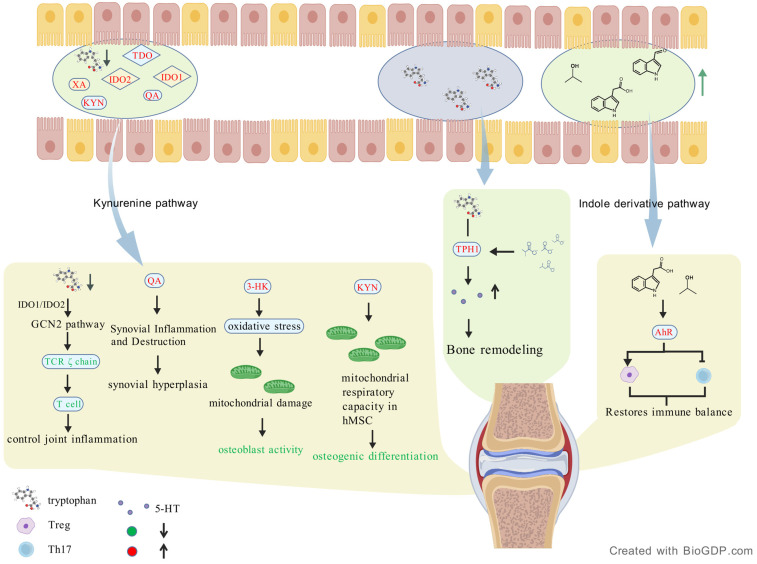
Tryptophan metabolism and its osteoimmune regulatory roles in RA. Gut dysbiosis in RA alters Trp metabolism, leading to reduced Trp levels and increased KYN, QA, and XA through the kynurenine pathway. Trp depletion activates the GCN2 pathway, suppressing TCR ζ-chain signaling and dampening T cell responses, thereby alleviating joint inflammation. QA promotes synovial inflammation and tissue destruction, while 3-HK induces oxidative stress and mitochondrial dysfunction, impairing osteoblast activity. In contrast, KYN enhances mitochondrial respiration in hMSCs, promoting osteogenic differentiation. Trp is also converted into 5-HT via TPH1, contributing to bone remodeling; this process can be enhanced by activating THP1 by SCFAs, indicating cross-talk between Trp and SCFA pathways. Additionally, Trp-derived indole metabolites such as IAA and IPA activate the AhR, promoting Treg differentiation, inhibiting Th17 responses, and restoring immune balance in RA. Created with BioGDP.com [33].

Taken together, Tryptophan-mediated gut–joint axis regulates immune responses through the KP and indole derivatives, affecting inflammation and joint damage in RA. Additionally, a small proportion of tryptophan (1–2%) is converted into serotonin in enterochromaffin cells through tryptophan hydroxylase 1 (TPH1), the expression of which is regulated by SCFAs. While the role of gut-derived serotonin in RA remains to be fully elucidated, serotonin is thought to play a role in controlling bone remodeling. This link between tryptophan, SCFAs, and serotonin highlights the role of gut metabolites in the gut–joint axis, influencing RA pathogenesis [94,95] (Figure 3).

### 2.2. Histamine Modulates Joint Dysfunction and Pain in RA

Emerging evidence suggests that histamine plays a critical role in the pathogenesis of RA, particularly in promoting joint inflammation and bone destruction. Immunohistochemical analyses have revealed increased expression of HDC, the key enzyme in histamine biosynthesis, in RA cartilage, along with the accumulation of histamine itself [15]. Histamine contributes to osteoclastogenesis through the upregulation of RANKL in synovial and immune cells, which in turn promotes the differentiation and activation of osteoclast precursors, ultimately enhancing bone resorption in RA [96,97]. Elevated histamine levels have been detected in the SF of RA patients, and histamine exerts its effects primarily through peripheral H1R, H2R, and H4R receptors [15,98]. These receptors are expressed in various joint-resident cells, including synoviocytes, osteoclast precursors, and chondrocytes.

Within the articular cartilage, histamine influences chondrocyte behavior via its receptors. Stimulation of H1R enhances the production of prostaglandin E2 (PGE2), promoting chondrocyte proliferation and inflammatory signaling [99]. H2R, on the other hand, modulates intracellular cAMP levels and may indirectly participate in inflammatory pathways [100]. Moreover, histamine induces the expression of matrix metalloproteinases (MMP-3 and MMP-13) via H1R activation, leading to extracellular matrix degradation. MMP-13 primarily targets type II collagen, while MMP-3 degrades proteoglycans and other matrix components, both contributing to cartilage erosion [101]. In addition, histamine modulates cytokine cascades through H2R-mediated regulation of IL-18 [102], a pivotal pro-inflammatory cytokine involved in RA pathogenesis. H2R also mediated the effects of histamine on osteoclast differentiation. H4R activation promotes the release of leukotriene B4 (LTB4) from mast cells (MCs) [103], a potent neutrophil chemoattractant that intensifies synovial inflammation. Additionally, IL-6 and Th17-related cytokines have been shown to regulate H4R expression [15], suggesting a feedback loop that further amplifies histamine-mediated bone destruction and may serve as a potential therapeutic target. The presence of H4R in synovial tissues, blood vessels and endothelial cells of RA patients further underscores its role in local inflammation as well as osteoclastogenesis [15]. Histamine receptors (H1R and H2R) are also present on human monocytes and macrophages [13,104]. Studies have also demonstrated that histamine can enhance the proliferation and migration of fibroblasts by increasing intracellular calcium ion concentrations.

On the other hand, the joint pain associated with rheumatoid arthritis is due to joint inflammation, and augmented by structural joint damage. Histamine is reported to directly sensitize nociceptors around joints and facilitates pain transmission, or indirectly causes pain via H1R. We previously reported that histamine modulates acute pain by regulating voltage gated sodium channel (Na_v_1.8) of nociceptive neurons [105]. Our previous work also proposed that histamine in the central nervous system is analgesic, while in the periphery, via H1 receptors, has an algesic effect [106]. Furthermore, luminal conversion of l-histidine to histamine by hdc^+^ *L. reuteri* activates H2R in gut, resulting in suppression of acute inflammation within the mouse colon [107], while blocking H2R signaling enhanced proinflammatory responses of mature neutrophils and suppressed neutrophil maturation, accelerating the progression of inflammation-associated colonic tumorigenesis 34781022. Collectively, these findings highlight the multifaceted role of histamine in driving pain and joint pathology in RA, incorporating context-specific and receptor-dependent effects.

Moreover, two additional metabolites of the gut microbiota—SCFAs and tryptophan—are implicated in interactions with histamine. Based on these observations, we aim to review further the role of histamine and its receptor system in mediating and regulating the gut–joint axis, with a particular focus on its contribution to the pathophysiology of RA. Here, we will delve into the metabolic pathways of histamine and explore its effects on the gut–joint axis, focusing on its influence on RA synovium and joints through various cell types (such as MCs, basophils, etc.), as well as the associated signaling pathways and molecular mechanisms. This will provide further insight into the specific role of histamine in the pathogenesis of RA.

## 3. Histamine Is the Central Hub in the Microbiota–Gut–Joint Axis

### 3.1. Histamine and Gut Microbiota Interactions

Beyond its local effects within the joints, histamine may also contribute to the systemic pathophysiology of RA through its involvement in the gut–joint axis. While host MCs and basophils predominantly synthesize histamine—evidenced by increased HDC expression in the synovial tissues of RA patients—emerging evidence suggests that the gut microbiota plays a pivotal role in modulating systemic histamine levels. In particular, certain gut-resident bacteria possess the enzymatic machinery (HDC) required to convert dietary L-histidine into histamine, thereby shaping the host’s histaminergic tone. This highlights a bidirectional interaction whereby both host and microbial sources of histamine may act in concert to influence immune responses and disease progression in RA. Supporting this, Li et al. reported that both L-histidine and histamine levels are positively correlated with the abundance of *Lactobacillus* and negatively associated with *Bacteroides* and *Prevotellaceae*_UCG-001 [108], indicating that specific microbial taxa may regulate host histamine metabolism. *Lactobacillus*, in particular, has been shown to possess HDC [109,110,111]. Given that *Lactobacillus* is often elevated in RA patients, this may contribute to increased histamine production in the gut environment. In addition, Barcik et al. identified several bacterial species with high HDC activity, including *Morganella morganii*, *Escherichia coli*, *Hafnia alvei*, *Proteus vulgaris*, *Proteus mirabilis*, *Enterobacter aerogenes*, *Raoultella planticola*, *Raoultella ornithinolytica*, *Citrobacter freundii*, *Pseudomonas fluorescens*, and *Photobacterium damselae* [112]. Notably, among these taxa, *Escherichia* and members of the *Proteobacteria phylum* are known to be enriched in the gut microbiota of RA patients. These findings further support the notion that dysbiosis in RA may lead to excessive microbial-derived histamine production, thereby exacerbating systemic inflammation and joint pathology.

SCFAs may interact with histamine pathways and further modulate immune responses in RA. Iljazovic et al. demonstrated that colonization with *Prevotella intestinalis* leads to decreased SCFA production, particularly acetate, and a concomitant reduction in intestinal IL-18 levels under steady-state conditions [113]. Given that IL-18 and IL-12 can upregulate HDC activity in host tissues and thus enhance histamine synthesis [114], these observations suggest that gut microbiota may indirectly regulate histamine levels via SCFAs-mediated pathways. Additionally, SCFAs can modulate the composition and function of gut microbiota, thereby influencing Gut microbiota derived histamine production and metabolism. They are also known to exert immunomodulatory effects by suppressing MCs and basophil activation, which are primary sources of peripheral histamine release. Therefore, the interaction between gut microbiota and histamine not only reflects the complex network of microbial metabolites in RA pathophysiology, but also provides a broader perspective on the metabolic interplay along the gut–joint axis.

### 3.2. Gut Microbiota Primed Histamine Production and Release Amplify the Damage of Synovium and Joints of RA

MCs are one of the major sources of histamine in the synovium of RA patients. MCs are located at the boundary where B cells and T cells aggregate [115]. Their numbers are significantly increased compared to healthy individuals [116]. MCs degranulation and histamine release lead to localized tissue edema and matrix destruction. Toll-like receptors (TLRs) are widely distributed in MCs and intestinal epithelial cells [117], linking innate and adaptive immune responses through the activation of Myeloid Differentiation Factor 88 (MyD88) and matrix metalloproteinase (MMP) signaling pathways, thereby forming a defensive barrier. However, excessive activation of TLR signaling can lead to the overproduction of inflammatory factors, disrupting immune homeostasis, exacerbating damage to the synovium and joints, and increasing the risk of autoimmune diseases like RA [118]. TLR2 and TLR4, respectively, respond to stimuli from Gram-positive and Gram-negative bacteria, triggering MCs degranulation and the release of various inflammatory mediators such as histamine, TNF-α, and IL-1β, further promoting synovial inflammation and bone destruction [8]. Moreover, in the intestinal environment, specific gut microbiota and their metabolites directly or indirectly act on MCs. Research has shown that *Prevotella* can stimulate epithelial cells to produce IL-6 and TNF-α. IL-6 not only enhances the differentiation of CD34+ progenitor cells into MCs but also significantly increases their reactivity, leading to increased degranulation and cytokine release mediated by FcεRI. The hemolysin-producing *Escherichia coli* (Hly+ *E. coli*) stimulates intestinal MCs to release histamine through MAPK activation [119]. *Lactobacillus* strains inhibit MCs activation and histamine release by downregulating the high-affinity IgE receptor (FCER1A) and the HRH4 receptor [120]. Among them, *Prevotella* and *Lactobacillus* were increased in RA, and *Escherichia coli* was also increased in RA, but the specific changes in Hly+ *E. coli* have not been reported. Furthermore, intestinal MCs could recognize the LPS of Gram-negative bacteria, which can activate MCs, prompting degranulation and histamine release in response to infection and inflammation [119].

MCs and chondrocytes can generate histamine, indicating their potential synergistic roles in the inflammatory response in RA [121]. Previous studies have demonstrated that basophils express GPR41, and acetate significantly induces calcium influx in basophils in a GPR41-dependent manner. Concurrently, propionate and butyrate promote the expression of the activation marker CD69 and cytokine IL-13 in basophils, as well as IgE-mediated basophil degranulation, while inhibiting IL-4 secretion [122]. This suggests that SCFAs have regulatory roles in basophil survival, IL-4 and IL-13 production, and degranulation. Basophils can release histamine [123], and earlier, we have listed the gut microbiota that show conspicuous changes in abundance in RA patients and are capable of producing SCFAs. This allows us to establish the gut microbiota–SCFA–basophil–histamine metabolic pathway.

In addition to be produced and stored in granules by MCs and basophils, and rapidly released upon immune stimulation, histamine can be de novo synthesized in various cells at the site of inflammation through the enzyme HDC, which is responsible for histamine synthesis, and released without storage. Building on this understanding, subsequent investigations have revealed that the gut microbiota can regulate HDC activity through pro-inflammatory factors, thereby increasing the levels of histamine in local tissues.

The elevated levels of these pro-inflammatory cytokines enhance HDC activity [124], thereby increasing local histamine production and release. *Staphylococcus* species in the gut secrete staphylococcal enterotoxin A (SEA) [125], which induces the proliferation and activation of T cells, leading to the release of pro-inflammatory cytokines such as IL-1, IL-2, IL-6, IFN-α, and IFN-γ [126]. Similarly, *E. coli* activates T cell proliferation through the secretion of enterotoxins (ENT), resulting in the release of inflammatory cytokines [127]. As a Gram-negative bacterium, *E. coli*’s endotoxins exert potent pro-inflammatory effects, promoting the secretion of IL-6 and TNF-α [128]. Furthermore, *E. coli* can disrupt the intestinal mucosal barrier, causing dysbiosis and increasing gut permeability, which facilitates bacterial translocation. Certain bacteria and LPS can translocate from the gut into the bloodstream and subsequently reach the liver. Hepatic macrophages and other cells expressing Toll-like receptor 4 (TLR4) recognize LPS via the LPS/TLR4 pathway, initiating downstream immune signaling responses. This activation leads to the upregulation of transcription factors such as NF-κB and activator protein-1 (AP-1), which increases the production of inflammatory cytokines, including IFN-γ, TNF-α, IL-6, and IL-1 [129].

Conversely, certain gut microbiota in the body inhibits pro-inflammatory factors. For example, *Bifidobacterium* species can reduce serum levels of IL-1β, TNF-α, and IL-6, and *Lactobacillus* strains can suppress the expression of cytokines such as IL-6 and interleukin-17 (IL-17) [130,131]. Additionally, *Lactobacillus reuteri* has been shown to inhibit the production of pro-inflammatory cytokines by intestinal epithelial cells and monocytes, leading to reduced plasma levels of cytokines like keratinocyte chemoattractant, IL-6, and IL-22 [132]. This suppression of inflammatory mediators consequently downregulates HDC expression, thereby reducing histamine production.

Moreover, studies have identified a positive feedback loop involving FLS, immune cells, pro-inflammatory cytokines, and histamine in RA. Certain gut bacteria can activate immune cells, promoting the release of pro-inflammatory cytokines, which in turn enhance HDC activity and histamine release. Histamine further stimulates FLS proliferation and the secretion of pro-inflammatory cytokines, exacerbating synovial inflammation and joint damage. FLS can produce various cytokines, including ILs, IFN, TNF, colony-stimulating factors, growth factors, and chemokines, which further enhance HDC activity, promoting histamine release [128]. FLS can also adhere to activated T cells, increasing the expression of intercellular adhesion molecule-1 (ICAM-1) and vascular cell adhesion molecule-1 (VCAM-1). ICAM-1, via the H2-cAMP-PKA signaling pathway, leads to increased secretion of TNF-α, IFN-γ, and IL-6, which further upregulates HDC activity, resulting in elevated histamine levels [124]. This forms a positive feedback loop that continuously amplifies the pathological progression of RA.

**Figure 4:**
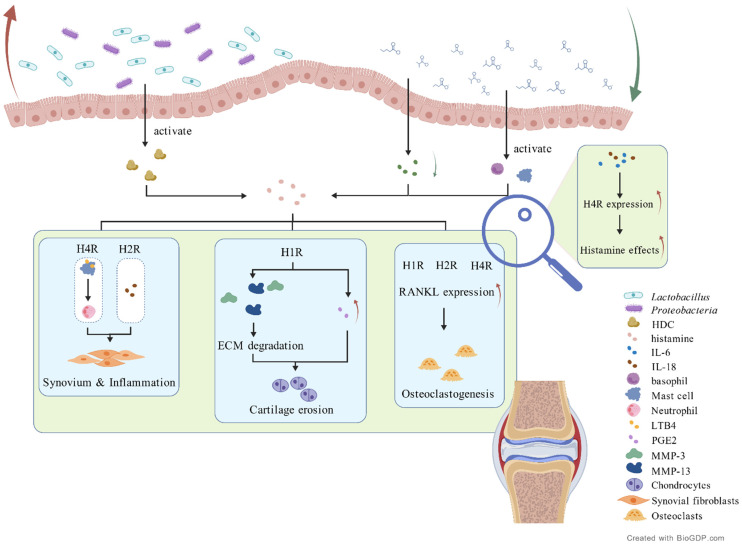
Histamine-mediated pathways linking gut dysbiosis to joint pathology in RA. Gut dysbiosis in RA, including overgrowth of *Lactobacillus* and *Proteobacteria*, increases histamine via microbial HDC, while SCFAs promote its release by activating MCs and basophils. Histamine enhances synovial inflammation through H4R-induced LTB4 production and neutrophil recruitment, and H2R-mediated IL-18 upregulation. In cartilage, H1R signaling increases PGE2 and induces MMP-3/13, driving ECM degradation and chondrocyte inflammation. Histamine also promotes osteoclastogenesis by upregulating RANKL in FLSs and immune cells, and modulates bone metabolism via H1R, H2R, and H4R, with IL-6/IL-18 further amplifying H4R expression under inflammatory conditions (Tables S1–S3). Created with BioGDP.com [33].

In addition to the aforementioned indirect pathways, certain gut microbiota species harboring HDC can directly enhance its histamine production. This aspect was previously discussed in the context of the gut–joint axis. Considering the alterations in microbiota composition in RA, significant fluctuations in the abundance of bacterial species such as *Escherichia coli*, *Morganella morganii*, *Proteus mirabilis*, and *Citrobacter freundii* have been observed, with these microbial shifts exerting a notable impact on histamine production. (Figure 4). As mentioned before, RA is accompanied by an imbalance of gut microbiota homeostasis. MCs and chondrocytes are excessively activated and release histamine, some of which accumulates in the mucosal tissue, while a portion enters the systemic circulation. When histamine reaches the joints via the bloodstream, it recruits immune cells, exacerbating the inflammatory response in the synovium and potentially attacking synovial fibroblasts, thereby intensifying local inflammation.

## 4. Discussion

Accumulating evidence now implicates that gut microbiota dysbiosis represents a critical driver of both the onset and progression of RA. Patients with RA typically display reduced microbial diversity accompanied by relatively consistent alterations in microbial community structure. At the phylum level, *Verrucomicrobia* and *Proteobacteria* are significantly enriched, whereas *Bacteroidetes* and *Firmicutes* are markedly depleted [17]. At the genus level, several studies have reported increased abundance of opportunistic pathobionts, including *Lactobacillus*, *Escherichia*, and *Prevotella*, alongside a pronounced reduction in classical anti-inflammatory commensals such as *Faecalibacterium*, *Roseburia*, and *Bifidobacterium* [18,19,20,21]. This flora imbalance is accompanied by elevated levels of systemic endotoxin (LPS) and pro-inflammatory factors (TNF-α, IL-1β, IL-6, CRP), which maintain a chronic inflammatory environment in the joints and drive disease progression [22]. Importantly, the expansion of *Proteobacteria*—particularly *Escherichia* [23] and *Pseudomonas* [24]—has been repeatedly detected in inflamed joints, implicating Gram-negative bacteria as potential direct contributors to RA pathogenesis [23,25,26,27]. Mechanistic investigations have further substantiated these associations: *Prevotella copri* promotes Th17/IL-17–mediated immune responses and exacerbates synovitis and bone destruction [30], whereas depletion of *Faecalibacterium prausnitzii* compromises immunoregulation and drives aberrant activation of fibroblast-like FLS [31]. Collectively, these changes accelerate osteoclast differentiation and bone erosion, highlighting the capacity of gut microbiota to orchestrate joint pathology through immune imbalance, FLS activation, and bone metabolic disruption [32].

Beyond alterations in microbial community profiles, gut microbiota–derived metabolites—including SCFAs, tryptophan derivatives, and histamine—are now regarded as central mediators bridging dysbiosis with RA pathophysiology. SCFAs such as acetate, propionate, and butyrate maintain intestinal barrier integrity, regulate immune responses, and influence osteoclast activity, thereby exerting bidirectional effects on both inflammation and bone metabolism. Notably, butyrate and propionate suppress TRAF6- and NFATc1-dependent signaling pathways, inhibit osteoclastogenesis, and attenuate joint erosion [48,50,133]. Tryptophan metabolism contributes additional layers of regulation: the kynurenine pathway and indole derivatives give rise to diverse immunomodulatory molecules, whereby IDO1 and quinolinic acid are linked to oxidative stress and aggravated synovitis, while indole derivatives such as IAA and IPA promote Treg differentiation, suppress Th17 responses, and confer joint protection [82]. Histamine, another key microbial metabolite, exerts pleiotropic actions on synovial fibroblasts, macrophages, osteoclasts, and chondrocytes through its receptors (H1R, H2R, and H4R), amplifying synovial inflammation, promoting osteoclastic bone resorption, and accelerating cartilage degradation. Overall, these metabolites not only play dynamic regulatory roles in the microbiota–gut–joint axis but also have double-edged effects in immunomodulation, synovial remodeling, and bone metabolism in RA, which not only reveal the potential pathogenic mechanisms but also provide new therapeutic opportunities for metabolic pathway intervention.

Emerging evidence further suggests that histamine may function as a central “hub” molecule linking microbial metabolites with host immune responses. Upstream, SCFAs profoundly reshape tryptophan metabolic flux. On the one hand, they upregulate TPH1 in enterochromaffin cells, thereby promoting the conversion of tryptophan into serotonin [94,95,134]. On the other hand, SCFAs inhibit IDO1 activity through two distinct mechanisms: by downregulating STAT1 to suppress IFN-γ–dependent IDO1 transcription, and by inhibiting histone deacetylases (HDACs) to reduce the production of proinflammatory cytokines such as TNF-α, IFN-γ, and IL-6, thereby further limiting IDO1 activation [135]. These effects collectively decrease kynurenine pathway flux while favoring the generation of microbiota-derived indole derivatives. Conversely, reduced SCFA levels are associated with impaired intestinal IL-18/IL-12 signaling, which indirectly releases the suppression of host HDC and enhances histamine synthesis [136]. Moreover, SCFAs exert cell–type–specific effects on histamine release: in MCs, propionate and butyrate suppress survival and histamine secretion through HDAC inhibition, whereas in basophils, they promote CD69 expression and IgE-dependent degranulation while simultaneously impairing survival and IL-4 secretion. SCFA-mediated receptor signaling also contributes, as exemplified by GPR41-dependent Ca^2+^ influx induced by acetate in basophils. These heterogeneous mechanisms together shape the overall pattern of histamine production and release [122].

Downstream, histamine can be produced by host cells—including MCs, basophils, and other tissue-resident populations—or by gut bacteria harboring HDC. Once released, it acts through H1R, H2R, and H4R expressed on fibroblast-like synoviocytes, macrophages, osteoclasts, and chondrocytes, where it amplifies cytokine production, upregulates RANKL expression, and accelerates osteoclast differentiation and matrix degradation [15,96,101]. In this way, histamine not only senses metabolic alterations in the gut, as shaped by SCFAs and tryptophan metabolism, but also transmits these signals into the joint microenvironment, where it redefines the inflammatory threshold and disease trajectory. Collectively, these findings position histamine as an integrative mediator that connects SCFAs, tryptophan metabolites, and microbial signals into a coherent metabolic–immune network, thereby providing a mechanistic rationale for targeting histamine and its receptors in RA.

Despite the growing body of evidence, important limitations remain. Histamine has been implicated in RA pathogenesis for decades. However, as of early 2026, clinical evidence for histamine-targeted interventions in RA remains limited. Early studies from the 1980s–1990s examined classical H1 and H2 receptor antagonists. In a small open-label investigation, Permin et al. (1981) administered cimetidine (H2 antagonist) plus mepyramine (H1 antagonist) to 12 patients with active RA, resulting in clinical improvement in six cases but deterioration in four, underscoring inconsistent and unpredictable effects [137]. A 2024 case report documented dramatic, repeatable joint pain relief with second-generation H1 antagonist fexofenadine as adjunctive therapy in a patient with refractory RA who could not tolerate conventional DMARDs or biologics [138]. This led to an ongoing Phase 1/2 randomized, placebo-controlled trial (NCT05264025), evaluating fexofenadine in active RA. Another concerted clinical development focused on selective H4 receptor (H4R) antagonism. Toreforant (JNJ-38518168), an oral selective H4R antagonist, was the lead compound to reach Phase II in RA patients with inadequate response to methotrexate (MTX). However, the Phase IIa study was terminated prematurely after one unrelated serious adverse event (secondary hemophagocytic lymphohistiocytosis); post hoc analyses nevertheless indicated modest reductions in RA signs and symptoms. The further larger Phase IIb dose-ranging study (3, 10, or 30 mg daily, n = 272) failed to meet the primary endpoint of change in DAS28-CRP at Week 12 [139,140]. Taken together, most current findings are based on cross-sectional associations rather than longitudinal or mechanistic studies, leaving the causal role of histamine in RA unresolved. Moreover, histamine receptor signaling is highly cell type and context specific, at times exerting opposing effects, which raises concerns that indiscriminate modulation may provoke excessive immunosuppression or unintended inflammation.

In addition, the crosstalk between histamine and other key microbial metabolites, such as SCFAs and indole derivatives, is still insufficiently characterized, particularly regarding their integrated actions on joint-resident immune and stromal cells. Future work should combine longitudinal cohorts, single-cell multi-omics, and humanized animal models to establish causality and dissect receptor- and environment-specific mechanisms. First, genetically engineered conditional knockdown or knockout mouse models (e.g., HDC^−/−^, Hrh2^−/−^ or H4R*^flox^*^/*flox*^ mice) are essential and helpful to delineate the crucial role of histamine in the modulation of dysbiosis in RA. Second, in vitro co-culture systems of intestinal epithelial cells, immune cells or microbiota-derived derivatives could reveal how histamine amplifies or antagonizes immune responses, depending on different receptors. Finally, longitudinal studies tracking histamine levels and microbial composition in RA cohorts would clarify temporal relationships between histamine fluctuations and disease activity, while metabolomic profiling of gut and joint tissues could uncover novel histamine-metabolite interaction networks.

Clinically, histamine levels and receptor profiles in synovial fluid or serum—integrated with microbiome signatures—may serve as composite biomarkers to complement DAS28, CRP, and ESR, thereby refining disease stratification and treatment monitoring. Furthermore, the interconnected tryptophan–SCFA–histamine axis offers opportunities for combined interventions, such as dietary or probiotic modulation alongside receptor antagonists, enabling multi-layered regulation from metabolic inputs to effector pathways. These strategies align with the immunometabolic basis of RA and underscore the translational potential of the histamine-mediated microbiota–gut–joint axis for precision therapy.

## 5. Conclusions and Future Perspectives

**Figure 5:**
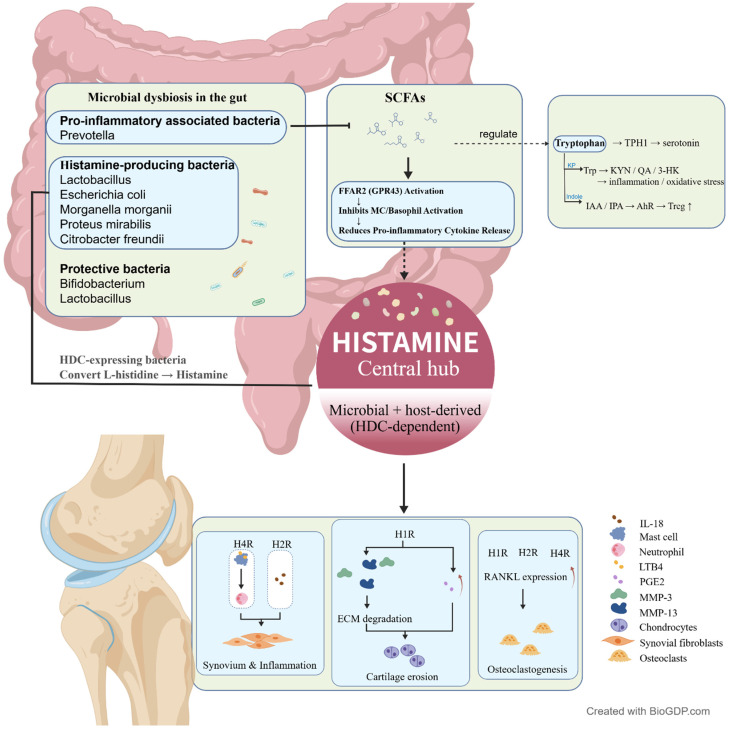
Histamine as a potential central mediator linking gut microbiota–derived metabolites with immune dysregulation and joint pathology in RA. Microbial alterations regulate histamine synthesis through histamine-producing bacteria (HDC-dependent), or indirectly via short-chain fatty acids and tryptophan metabolites that modulate mast cell activation, epithelial barrier integrity, and inflammatory signaling. Histamine, acting through H1R, H2R, and particularly H4R, promotes synovial fibroblast activation, cytokine production, and osteoclastogenesis, thereby amplifying synovitis and bone erosion in RA. Created with BioGDP.com [33].

Collectively, current evidence supports histamine as a potential central mediator linking gut microbiota–derived metabolites with immune dysregulation and joint pathology in RA. Microbial alterations influence histamine synthesis directly through histamine-producing bacteria, or indirectly via short-chain fatty acids and tryptophan metabolites that modulate mast cell activation, epithelial barrier integrity, and inflammatory signaling. Histamine, acting through H1R, H2R, and particularly H4R, promotes synovial fibroblast activation, cytokine production, and osteoclastogenesis, thereby amplifying synovitis and bone erosion (Figure 5). However, most mechanistic insights are derived from animal and in vitro studies, and direct clinical and molecular validation in patients remains limited. Future work integrating longitudinal human cohorts, multi-omics approaches, and receptor-targeted interventions will be essential to clarify causality and evaluate the therapeutic potential of targeting the histamine-centered microbiota–gut–joint axis in RA.

## Data Availability

No new data were created or analyzed in this study. Data sharing is not applicable to this article.

## Supplementary Materials

The following supporting information can be downloaded at: https://www.mdpi.com/article/10.3390/ijms27052315/s1.
