# Supplementary material for: A Potential Central Hub of Histamine in the Microbiota–Gut–Joint Axis in Rheumatoid Arthritis: Mechanisms and Translational Implications
Source: Int J Mol Sci. 2026 Mar 1;27(5):2315. doi: 10.3390/ijms27052315 (PMC12984847; doi:10.3390/ijms27052315)
Supplement: Supplementary file 1 [file ijms-27-02315-s001.zip › ijms-4057742-supplementary.pdf]

Table S1. Expression and functions of histamine receptors in joint-resident and immune cells in RA

Receptor	Major cell types	Main signaling	Functional effects in RA	Pathological outcome
**H1R**	Chondrocytes, FLSs, sensory neurons, macrophages	Gq → PLC → Ca^{2+} ↑	↑ PGE2 production; ↑ MMP-3/13; ↑ inflammatory cytokines; nociceptor sensitization	Cartilage degradation, synovitis, pain
**H2R**	Monocytes/macrophages, osteoclast precursors, intestinal epithelial cells	Gs → cAMP ↑	Modulates IL-18; regulates immune activation; context-dependent anti/pro-inflammatory effects; influences osteoclast differentiation	Immune imbalance, bone remodeling
**H4R**	FLSs, osteoclasts, mast cells, neutrophils, synovial tissues, vasculature	Gi/o → MAPK/NF-κB	↑ chemotaxis; ↑ cytokine release; ↑ RANKL signaling; ↑ osteoclastogenesis; ↑ LTB4 release	Synovial inflammation, bone erosion (dominant pathogenic receptor)

Table S2. Key gut microbial taxa associated with SCFAs, indole derivatives, and histamine production in RA

Metabolite	Representative taxa	Change in RA	Mechanistic effects on host	Relevance to histamine axis
**SCFAs (butyrate, acetate, propionate)**	Faecalibacterium prausnitzii, Roseburia, Lachnospira, Ruminococcus, Bifidobacterium, Clostridiales	↓ (most butyrate producers)	↑ Tregs; ↑ barrier integrity; ↓ NF-κB; ↓ osteoclastogenesis	Suppress MC/basophil activation; indirectly reduce histamine release
**Indole derivatives (IAA, IPA, IAld)**	Lactobacillus spp., Clostridium spp., Peptostreptococcus, Bacteroides	↓	AhR activation; ↑ Tregs; ↓ Th17; antioxidant effects	Anti-inflammatory environment may counteract histamine signaling
**Histamine (HDC^{+} bacteria)**	Lactobacillus reuteri, Morganella morganii, Escherichia coli, Proteus spp., Hafnia alvei, Raoultella spp., Citrobacter freundii	↑ (Proteobacteria enrichment)	Direct histamine synthesis from L-histidine	↑ systemic histamine → activate H1R/H2R/H4R → joint inflammation
**LPS producers (pro-**	Proteobacteria, Escherichia,	↑	↑ TNF-α/IL-6/IL-1β → ↑ HDC	Indirectly enhance

**inflammatory)**	Pseudomonas	expression	histamine production

Table S3. Crosstalk among SCFAs, tryptophan metabolism, and histamine in the microbiota–gut–joint axis

Interaction	Mechanism	Consequence in RA
**SCFAs → histamine**	HDAC inhibition; suppress MC survival	↓ histamine release
**SCFAs → IDO1/Trp pathway**	↓ STAT1/IDO1 activity	Shift away from pro-inflammatory KP metabolites
**Dysbiosis → IL-18/IL-12 → HDC**	Cytokine-induced HDC activation	↑ histamine synthesis
**Histamine → FLS/osteoclasts**	↑ RANKL, ↑ cytokines	Bone erosion
**Histamine ↔ cytokines**	Positive feedback loop	Chronic inflammation amplification
